# Ceramides Profile Identifies Patients with More Advanced Stages of Colorectal Cancer

**DOI:** 10.3390/biom10040632

**Published:** 2020-04-19

**Authors:** Adam R. Markowski, Agnieszka U. Błachnio-Zabielska, Katarzyna Guzińska-Ustymowicz, Agnieszka Markowska, Karolina Pogodzińska, Kamila Roszczyc, Justyna Zińczuk, Piotr Zabielski

**Affiliations:** 1 Department of Internal Medicine and Gastroenterology, Polish Red Cross Memorial Municipal Hospital, 79 Henryk Sienkiewicz Street, 15-003 Bialystok, Poland; 2 Department of Hygiene, Epidemiology and Metabolic Disorders, Medical University of Bialystok, 2C Adam Mickiewicz Street, 15-222 Bialystok, Poland; agnieszka.blachnio@umb.edu.pl (A.U.B.-Z.); karolina.pogodzinska@umb.edu.pl (K.P.); 3 Department of General Pathomorphology, Medical University of Bialystok, 13 Jerzy Waszyngton Street, 15-269 Bialystok, Poland; 4 Department of Organic Chemistry, Medical University of Bialystok, 2A Adam Mickiewicz Street, 15-222 Bialystok, Poland; agnieszka.markowska@umb.edu.pl; 5 Department of Medical Biology, Medical University of Bialystok, 2C Adam Mickiewicz Street, 15-222 Bialystok, Poland; kamila.roszczyc@gmail.com (K.R.); piotr.zabielski@umb.edu.pl (P.Z.); 6 Department of Clinical Laboratory Diagnostics, Medical University of Bialystok, 15A Jerzy Waszyngton Street, 15-269 Bialystok, Poland; justyna.zinczuk@umb.edu.pl

**Keywords:** colorectal cancer, advanced stage, ceramides profile

## Abstract

Much attention is paid to different sphingolipid pathways because of their possible use in diagnostics and treatment. However, the activity status and significance of ceramide pathways in colorectal cancer are still unclear. We analyzed colorectal cancer patients to evaluate sphingolipid profiles in the blood, colorectal cancer (CRC) tissues, and healthy surrounding colorectal tissues of the same patient, simultaneously, using liquid chromatography coupled with triple quadrupole mass spectrometry. Furthermore, we measured protein expression of de novo ceramide synthesis enzymes and mitochondrial markers in tissues using western blot. We confirmed the different sphingolipid contents in colorectal cancer tissue compared to healthy surrounding tissues. Furthermore, we showed changed amounts of several ceramides in more advanced colorectal cancer tissue and found a prominently higher circulating level of several of them. Moreover, we observed a relationship between the amounts of some ceramide species in colorectal cancer tissue and plasma depending on the stage of colorectal cancer according to TNM (tumors, nodes, metastasis) classification. We think that the combined measurement of several ceramide concentrations in plasma can help distinguish early-stage lesions from advanced colorectal cancer and can help produce a screening test to detect early colorectal cancer.

## 1. Introduction

Every year, colorectal cancer (CRC) is diagnosed in over a million people worldwide [1]. A continuous increase in morbidity is observed in many countries, including Poland, where CRC kills 40 people every day. Stable or decreasing trends are only observed in countries with the highest social and economic development indexes [2]. This reduction in mortality is probably due to improved access to endoscopy and improvements in specialized surgical and oncological care. Early diagnosis is crucial, as early colorectal cancer is fully curable, while the management of the more advanced disease is associated with increased morbidity. Screening programs for early detection of CRC with colonoscopy make it possible to diagnose a larger number of CRCs during the first stages of cancer development, with a better chance of ultimate cure. Since colorectal cancer is a very serious clinical problem and an effective treatment method is still lacking, it is highly important to know all aspects of its cell metabolism to discover new diagnostic and therapeutic targets.

Among the thousands of different lipids examined during experimental apoptosis, accumulation of sphingolipids accounted for almost 90% of the changes in a human CRC cell line [3]. Ceramides are the central bioactive molecules in sphingolipid metabolism. The content of ceramides in a cell depends on the balance between the rates of their production and degradation [4]. These compounds can be synthesized by multiple pathways [5]. De novo synthesis begins with the condensation of serine and palmitoyl-CoA, finally leading to the formation of ceramide by the action of serine-palmitoyltransferase (SPT), 3-ketosphingosine reductase, ceramide synthase (CerS), and dihydroceramide desaturase. Each CerS utilizes a fatty acyl-CoAs with a defined acyl chain length; it also exhibits differential tissue distribution. Intermediary products of this pathway are 3-ketodihydrosphingosine, sphinganine (SPA), and dihydroceramide [5]. Another method of ceramide production is the hydrolysis of sphingomyelin (SM), catalyzed by neutral and acid sphingomyelinase. Degradation of glycosphingolipids by the action of hydrolases is another source of ceramide (Cer). In the salvage pathway, hydrolytic enzymes, including sphingosine-1-phosphate phosphatase (S1PP), and ceramidases, produce sphingosine and Cer.

Ceramides are essential components of cell membranes, and in response to physiological or stress stimuli, they undergo rapid turnover, which substantially changes their biophysical properties. Furthermore, ceramides participate in intracellular signaling pathways as key mediators of various cellular processes. They are involved in cell migration and adhesion, aging, inflammatory response, proliferation, and differentiation. In addition, ceramides have also been shown to be involved in growth inhibition and apoptosis [6,7]. However, some sphingolipids, such as sphingosine-1-phosphate (S1P) play the opposite role in the cell. The diversity of cellular responses mediated by sphingolipids results from the fact that these molecules regulate the activity of many different enzymes, including kinases, phosphatases, and lipases [8].

Two primary pathways lead to apoptosis. Induction of the intrinsic (mitochondrial) pathway is activated in response to cellular stresses like nutrient deprivation, hypoxia, or DNA damage. In turn, the extrinsic pathway can be initiated by activation of death receptors located on the cell surface, by binding with their specific ligands. Cancer cells engage various mechanisms to evade programmed cell death, and the intrinsic pathway of apoptosis is frequently disrupted in cancer cells [9].

Accumulating evidence suggests that ceramides are also implicated in cancer development and progression. Defects of ceramide metabolism in cancer cells can contribute to their abnormal proliferation and survival. The available data indicates that their metabolism is altered in various types of cancer [10]. Considering that ceramides inhibit the expression of matrix proteolytic enzymes called metalloproteinases, which play a key role in tumor invasion and metastasis, it is suggested that ceramides may thus inhibit cancer progression [11]. However, the mechanisms by which sphingolipids contribute to the formation of cancer are still unclear.

The current cancer clinical evaluation is based on the TNM (tumors, nodes, metastasis) classification system and does not always reflect the different biological behavior of cancers at the same stage. Further new indicators are constantly being sought that may be useful for better colorectal cancer staging; ceramide profile may become one of them. With the prevalence of screening programs, the percentage of patients diagnosed with early cancer increases, so additional classification categories are important to precisely define the diagnosis and determine the best therapeutic management.

Therefore, the objective of the study was to perform a comparative analysis of sphingolipid profiles in CRC tissue and normal colorectal tissue, as well as in the blood, and to broaden the knowledge on the role of sphingolipids in the formation of cancer. In addition, the purpose was to identify those ceramides in the blood that could serve as a biomarker for colorectal cancer.

## 2. Materials and Methods

### 2.1. Patients

The study was in line with the principles outlined in the Declaration of Helsinki and approved by the Ethical Committee for Human Studies of the Medical University of Bialystok, Poland, ethics committee approval no R-I-002/228/2018. All patients gave their written informed consent for participation in the study. The present study included 45 consecutive adult patients with primary CRC who qualified for curative surgery. Pre-treatment diagnosis of CRC was evaluated based on colonoscopy with biopsy and CT scan. Blood samples were taken before surgery during routine testing. A 10 mL of peripheral blood was collected for EDTA (ethylenediaminetetraacetic acid) probes from each patient. The blood was centrifuged for 15 min, then plasma samples were separated and frozen at −80 °C. Tumor and surrounding normal tissue samples were obtained intraoperatively. The taken samples for the biochemical studies had their representation in a histological assessment, which was confirmed using the intraoperative frozen sections. The dissected tissues were immediately placed into liquid nitrogen and transferred for histological evaluation. Pathologic analysis using a standardized reporting template was performed on the resected specimens and staged according to the American Joint Committee on Cancer criteria, version 8 guidelines. The patients were classified according to the TNM staging (tumors, nodes, metastasis). All participants were of Caucasian descent. Rectal cancer patients received preoperative radiotherapy.

### 2.2. Sphingolipid Content in Tissues and Plasma

Sphingolipid content was measured using the ultra-high-performance liquid chromatography coupled with triple quadrupole mass spectrometry (UHPLC/MS/MS) approach according to Blachnio-Zabielska et al. [12]. Briefly, tissue samples (20 mg) were pulverized, then homogenized in a solution composed of 0.25 M sucrose, 25 mM KCl, 50 mM Tris, and 0.5 mM EDTA, pH 7.4. Immediately afterwards, 50 μL of the internal standard solution (ISTD solution; 17C–sphingosine and 17C–S1Pd17:1/8:0, d17:1/18:0, d17:1/18:1(9Z), d17:1/20:0, d17:1/24:0, and d17:1/24:1(15Z) (Avanti Polar Lipids, Alabaster, Al) as well as 2 mL of an extraction mixture (isopropanol:water:ethyl acetate, 30:10:60; *v:v:v*) were added to each homogenate. For plasma samples [13], the homogenization step was omitted, and ISTD solution and extraction buffer was added directly to 50 μL of plasma. The mixture was vortexed, sonicated, and then centrifuged for 10 min at 4000 rpm, 4 °C (Sorvall Legend RT). The supernatant was transferred to a new vial and the pellet was re-extracted with 2 mL of the extraction mixture. After centrifugation, the supernatants were combined and evaporated under nitrogen. The dried sample was reconstituted in 100 μL of LC Solvent B (2 mM Ammonium formate, 0.1% formic acid in methanol) for liquid chromatography tandem mass spectrometry (LC/MS/MS) analysis. Sphingolipids were analyzed using a Sciex Qtrap 6500 + triple quadrupole mass spectrometer (SCIEX, Framingham, MA, USA) using a positive ion electrospray ionization (ESI) source (except S1P, which was analyzed in negative mode) with multiple reaction monitoring (MRM) against standard curves constructed for each analyzed compound. Chromatographic separation was performed using Shimadzu ultra-high-performance liquid chromatography (UHPLC) (Shimadzu, Kyoto, Japan) (Manufacturer, City, State abbrev., if USA or Canada, Country) on Zorbax SB-C8 column 2.1 × 150 mm, 1.8 μm, with the use of binary gradient (1 mM ammonium formate, 0.1% formic acid in water as Solvent A, and 2 mM ammonium formate, 0.1% formic acid in methanol as Solvent B, at a flow rate of 0.4 mL/min).

### 2.3. Western Blot 

A total of 22 samples from 11 subjects (tumor and adjacent normal tissue) were used for estimation of protein expression of selected enzymes of sphingolipid metabolism. Equal amounts of protein (15 µg) from tumor and normal tissue were separated using SDS–PAGE (Criterion TGX 10% precast gels, Criterion Cell electrophoresis equipment, Bio-Rad, Hercules, CA, USA). The separated proteins were transferred onto polyvinylidene difluoride (PVDF) membranes (TransBlot Turbo semi-dry transfer system, Bio-Rad). After blocking in Tris-buffered saline with 5% nonfat dry milk, the membranes were incubated with an appropriate primary antibody. The following target proteins were quantified using primary antibodies: serine palmitoyltransferase (SPT) (SPTLC2 catalytical subunit, ab23696, Abcam, Cambridge, UK), ceramide synthase 1 (CerS1, ab98062, Abcam), ceramide synthase 2 (CerS2, ab85567, Abcam), ceramide synthase 5 (CerS5, ab73289, Abcam), and cytochrome c oxidase subunit IV (COX IV, #4844, Cell Signaling, Danvers, MA, USA). After incubation with appropriate horseradish peroxidase (HRP)-conjugated secondary antibody, protein bands were visualized with the use of a Bio-Rad ChemiDoc XRS+ system. The protein expression values were normalized to glyceraldehyde 3-phosphate dehydrogenase loading control (GADPH, ab9485, Abcam), measured from parallel runs on separate gel, and expressed as fold changes over control group values. Unless stated otherwise, all the chemicals and equipment used for immunoblotting were purchased from Bio-Rad. 

### 2.4. Statistical Analysis

Comprehensive data were processed using Statistica 13.3 (TIBCO Software Inc., Palo Alto, CA, USA). and shown as the mean (M) ± standard error (SE). The mean values of western blot proteins in tumor samples and normal tissues were compared using a paired t-student test. Descriptive analysis, difference analysis, variance analysis, and logistic regression analysis were used to identify the relevant variables related to more advanced colorectal cancer. Those features that were found significant were further investigated to discover their prediction capability. To evaluate the diagnostic power of the devised sphingolipids panel, receiver operating characteristic curves (ROC) were constructed, the area under the curve (AUC) was calculated, and the optimal cut-off value and Youden index were determined. Statistical significance was assumed if a p-value was less than 0.05.

## 3. Results

### 3.1. Patient Demographics

Of the 45 patients (Table 1), 12 (26.7%) were female. The mean age of CRC patients at diagnosis was 67.1 ± 1.9 years (range 35–90). Depending on the gender, the age was 67.8 ± 3.9 years (range 48–87) and 66.9 ± 2.2 years (range 35–90), for women and men, respectively. Of the colorectal carcinomas, 31.1% (*n* = 14) were located in the rectum, 22.2% (*n =* 10) in the sigmoid colon, 13.3% (*n =* 6) in the cecum, and 33.3% (*n =* 15) in other parts of colon. The average age of patients with the tumor located in several parts of the large intestine was 63.4 ± 3.8 years (range 35–90), 66.2 ± 3.2 years (range 53–87, 70.0 ± 3.0 years (range 48–87), and 70.1 ± 5.9 years (range 45–84), for rectum, sigmoid colon, cecum, and other parts of the colon, respectively. The number of patients in each group according to TNM classification was as follows: *n =* 14 (TNM I), *n =* 10 (TNM II), *n =* 15 (TNM III), *n =* 6 (TNM IV). Depending on the progression and stage of the disease (TNM I+II vs. TNM III+IV), the average age was 68.9 ± 2.6 years and 66.1 ± 2.7 years, respectively. The group of patients with well-differentiated cancer (low grade, G1) was the least numerous (*n =* 2), and the group of patients with moderately differentiated cancer (intermediate grade, G2) was the most numerous (*n =* 37). Distant metastases were found in 6 patients. Most patients (*n =* 24) had no evidence of lymph node metastases.

### 3.2. Pathologic Overview

Mucinous histology (>50% mucinous component) was found in 17.8% of tumors. Patients with stage I, II, III, and IV disease according to TNM-8 represented 29.6%, 22.7%, 34.1%, and 13.6% of cases at the time of diagnosis, respectively. In the vast majority of patients (95.4%), the depth of tumors was T2 and T3, i.e., 34.0%, and 61.4% of all cases, respectively. Of the cases, 2.3% had early-stage (T1) lesions, and 13.6% of CRC patients had distant metastases, while 40.9% had lymph node involvement (N1—25%, N2—15.9%). All patients were white; most patients (84.1%) had a moderately differentiated tumor, while 13.6% had poorly differentiated CRC. Depending on the stage (TNM I+II and TNM III+IV), the average maximum tumor size was 46.74 ± 5.01 mm and 49.75 ± 5.42 mm, respectively, and did not differ statistically between groups. 

### 3.3. Content of Sphingolipids in Tissues

The average sphingolipid content in tissues expressed as pmol per 1 mg of tissue, is shown in Table 2. Of the sphingolipids tested, by far the highest contents in CRC tissue were found for C16:0-Cer (120.81 pmol/mg, 80.4% of total ceramide) and C24:1-Cer (14.00 pmol/mg, 9.3% of total ceramide), which was similar to normal intestinal tissue (104.05 pmol/mg, 78.25% of total ceramide, and 14.06 pmol/mg, 10.6% of total ceramide, respectively).

Simultaneously, of the remaining sphingolipids, the highest content (distinct than in normal tissue) was demonstrated for C24:0-Cer (6.55 pmol/mg vs. 4.77 pmol/mg; *p* < 0.0013) and Sph (6.38 vs. 2.37, *p* < 0.001). Sphingolipid contents in tumor and normal intestinal tissue were compared. CRC tissue also showed an increased amount of S1P (0.05 vs. 0.02; *p* < 0.00013), SPA (1.48 vs. 0.72; *p* < 0.0003), and C14:0-Cer (1.60 vs. 1.15; *p* < 0.0031), compared to normal intestinal tissue. At the same time, tumor tissue was found to have a significantly lower C18:0-Cer content (3.09 vs. 4.07; *p* < 0.0039) and C20:0-Cer content (0.88 vs. 1.33; *p* < 0.00001) than in normal colorectal tissue, Table 2. 

We examined the relationship of sphingolipid levels in CRC tissue on tumor localization at four different parts of the large intestine. The relationships were complex and ambiguous. But the amount of total ceramides was the lowest in sigmoid and cecum tumors (121.69 ± 21.36 and 128.11 ± 22.82 respectively) and the largest in rectal tumors (182.17 ± 12.19); the difference in these cases was statistically significant (*p* = 0.016). In addition, the level of remaining sphingolipid species was the highest (S1P 0.08 ± 0.02, Sph 9.26 ± 2.57, SPA 2.40 ± 0.70) in one location (cecum) and the lowest in the sigmoid colon (S1P 0.03 ± 0.004) or in other parts of the large intestine, except for the rectum, sigmoid colon, and the cecum (Sph, SPA). The difference was statistically significant only for S1P (*p* = 0.03).

A different sphingolipids profile was found in normal intestinal tissues; of the remaining sphingolipids, the highest content was demonstrated for C18:0-Cer (4.07 pmol/mg) and C24:0-Cer (4.77 pmol/mg).

### 3.4. Concentration of Sphingolipids in the Plasma

The plasma profile of sphingolipids was different than in tissues. By far the highest concentration in the plasma was found for C24:0-Cer (2502.77 pmol/mL; 47.4% of total ceramide) and C24:1-Cer (1474.22 pmol/mL; 27.9% of total ceramide). A somewhat smaller, but still significant, concentration was demonstrated for C22:0-Cer, C16:0-Cer, and S1P.

### 3.5. Western Blot

**Figure 1:**
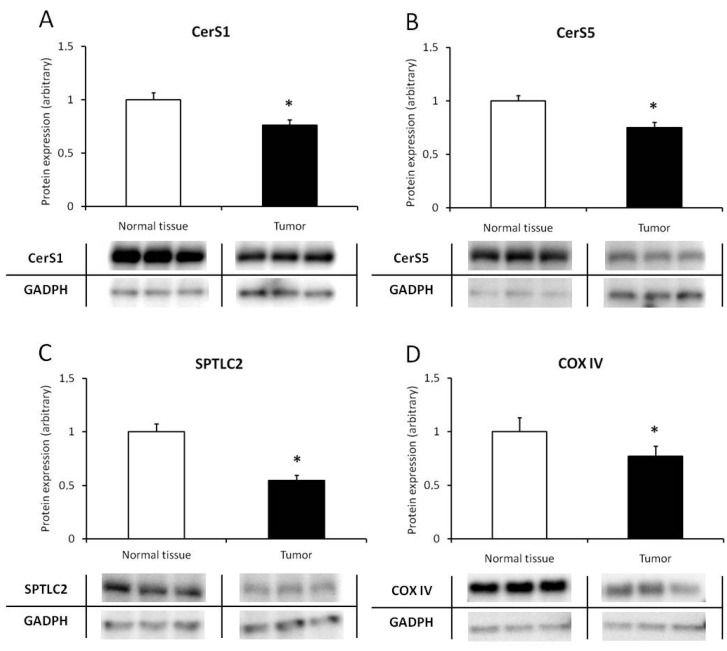
Protein expression of de novo ceramide synthesis enzymes (**A**)–(**C**) and mitochondrial marker (**D**) in normal colorectal tissue and colorectal cancer tissue. The values represent the mean protein expression +/- SEM, normalized to glyceraldehyde 3-phosphate dehydrogenase (GADPH) housekeeping protein expression; * *p* < 0.05 vs. tumor tissue (*n =* 11), as estimated by paired *t*-test. (**A**) CerS1–ceramide synthase 1; (**B**) CerS5–ceramide synthase 5; (**C**) SPTLC2–serine palmitoyltransferase catalytical subunit 2; (**D**) COX IV–cytochrome C oxidase, subunit IV; GADPH–glyceraldehyde-3-phosphate dehydrogenase. Pictures below graphs show representative blots from sample pools and were not used for calculation.

An analysis assessing the content of the enzymes involved in the de novo synthesis of ceramides gave an ambiguous result. The protein content was lower in CRC tissue compared to normal intestinal tissues, for serine palmitoyltransferase (1.0 ± 0.10 vs. 0.43 ± 0.06; *p* = 0.0003), ceramide synthase 1 (1.0 ± 0.07 vs. 0.73 ± 0.06; *p* = 0.002), ceramide synthase 5 (1.0 ± 0.05 vs. 0.71 ± 0.05; *p* = 0.00007), and cytochrome C oxidase IV (1.0 ± 0.13 vs. 0.77 ± 0.09; *p* = 0.041) (Figure 1). The decrease in the amount of ceramide synthase 2 protein (1.0 ± 0.083 vs. 0.89 ± 0.09; *p* = 0.2), although showing a similar trend, did not reach statistical significance.

### 3.6. Sphingolipids in Advanced Colorectal Cancer

Not all analyzed plasma sphingolipids showed gradual changes that directly correlated with the increase of the CRC stage. This was in the case of SPA, C14:0-Cer, C22:0-Cer, C24:0-Cer, and C24: 1-Cer, while Sph, S1P, and especially C16:0-Cer, C18:0-Cer, and C20:0-Cer, which exhibited a clearly progressive and unequivocal increase in line with the rise in TNM grade. According to these observations, the grade of CRC classification in the studied patient population does not correspond exactly to changes in sphingolipid metabolism in each case. Therefore, to capture and visualize such changes, groups were combined for statistical analysis and this procedure allowed us to observe the interdependencies presented in this manuscript.

When patients were divided into two groups depending on the severity of CRC according to TNM (TNM I+II vs. TNM III+IV), a higher tumor content of C20:0-Cer and C24:1-Cer in the advanced (TNM III+IV) group was observed (1.03 ± 0.09 vs. 0.78 ± 0.09, *p* = 0.0432; and 15.02 ± 1.01 vs. 13.47 ± 1.30, *p* = 0.0478; respectively) (Table 3). A similar trend was observed for C18:0-Cer, but it did not reach statistical significance (3.52 ± 0.31 vs. 2.79 ± 0.23, *p* = 0.0721).

Furthermore, the higher plasma concentration of C16:0-Cer, C18:1-Cer, and C20:0-Cer in the group with a higher stage of neoplastic disease (TNM III+IV) were observed (522.08 ± 41.32 vs. 402.40 ± 29.17, *p* = 0.0186; 7.44 ± 0.79 vs. 5.74 ± 0.24, *p* = 0.0317; and 56.45 ± 3.96 vs. 45.91 ± 3.09, *p* = 0.0452; respectively), Table 4. A similar trend was observed for C24:1-Cer, but it did not reach statistical significance (1608.57 ± 131.28 vs. 1335.95 ± 89.65, *p* = 0.0519).

Plasma C24:1-Cer, C18:1-Cer, and C16:0-Cer positively correlated with CRC stage according to TNM. (*R* = 0.32, *R* = 0.35, and *R* = 0.37, respectively). A similar relationship was found for C20:0-Cer, C18:1-Cer, and C16:0-Cer and lymph node involvement (*R* = 0.42, 0.37, and 0.38, respectively), but not for T and M components or maximum tumor size and grading.

**Figure 2:**
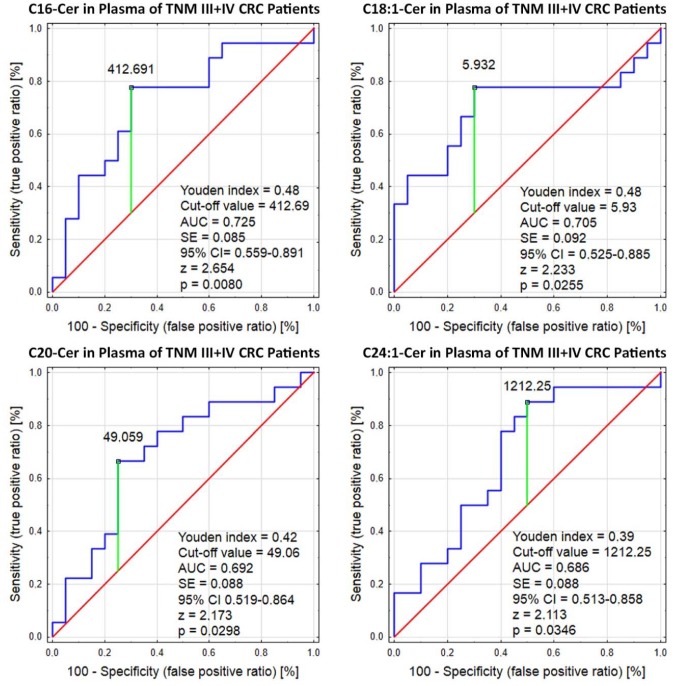
Receiver operator characteristic (ROC) curve analysis of statistically significant plasma sphingolipids (C16:0-Cer, C-24:1-Cer, C18:1-Cer, and C20:0-Cer) in patients with advanced CRC (TNM III+IV).

To detect and identify potential biochemical biomarkers associated with advanced CRC that could potentially be suitable for non-invasive diagnostics, plasma sphingolipids concentration was further analyzed. Our observations indicate that concentrations of four ceramides (C16:0-Cer, C18:1-Cer, C20:0-Cer, and C24:1-Cer) significantly change in the plasma of patients with advanced CRC (Figure 2). The AUC for C16:0-Cer was 0.725 (*p* = 0.008), for C18:1-Cer was 0.705 (*p* = 0.0125), for C20:0-Cer was 0.692 (*p* = 0.029), and for C24:1-Cer was 0.686 (p = 0.034). The sufficient discrimination (AUC > 0.7) in CRC patients in TNM stages III+IV was only found for C16:0-Cer and C18:1-Cer. For the other two ceramides (C20-Cer, C24:1-Cer), discrimination was weak, although still statistically significant. This means that there is an over 70% chance that the model will be able to distinguish patients between early (TNM stage I+II) and advanced (TNM stage III+IV) CRC. Therefore, a significantly higher risk of advanced colorectal cancer was demonstrated when plasma concentration of C16:0-Cer was higher than 412.61 pmol/mL, while plasma concentration of C18:1-Cer was higher than 5.93 pmol/mL. 

## 4. Discussion

Sphingolipid metabolic pathways in a healthy cell are complex and not yet fully understood. Even less is known about altered sphingolipid metabolism in colorectal cancer cells. In the present study, we evaluated the profile of ceramides in colorectal cancer tissue and healthy colorectal tissue from the areas surrounding a tumor to better understand sphingolipid metabolism in the development and progression of colorectal cancer. Furthermore, we explored the plasma ceramides profile in the same patients simultaneously, to reveal possible non-invasive diagnostic biomarkers.

The highest content in normal colorectal tissue was found for C16:0-Cer (78.25% of total ceramides) and C24:1-Cer (10.60% of total ceramides). These results are consistent with previous observations of other authors. Chen et al. also found the highest amount of C16:0-Cer and C24:0-Cer in human non-tumor colon tissues [14]. Among the evaluated ceramides in the current study, by far the highest content in CRC tissue was found for C16:0-Cer (80.36% of total ceramide), which was similar to normal colorectal tissue. Moreover, although C24:1-Cer amount in CRC did not differ statistically from normal tissue as well, the study showed that sphingolipid metabolism is to some extent changed, because we observed that ceramides tend to accumulate in CRC tissue. Of the remaining analyzed sphingolipids, the highest content (and higher than in normal tissue) was demonstrated for C24:0-Cer and Sph. CRC tissue also showed increased amounts of S1P, SPA, and C14:0-Cer. In turn, significantly lower C18:0-Cer and C20:0-Cer contents in the tumor were found otherwise. In our study, the difference in total ceramides amount between CRC tissue and adjacent healthy tissue did not reach statistical significance, but the growing trend in the tumor was clearly visible (150.34 pmol/mg vs. 132.90 pmol/mg) 

A different sphingolipid profile in colorectal cancer tissue than in normal intestinal tissues is consistent with the results of previous studies of other authors who revealed the presence of various distribution trends of individual ceramide analogs and showed that the level of some ceramide species (C16:0-Cer, C24:0-Cer, and C24:1) is increased, while the level of others (C18:0-Cer and C20:0-Cer) is reduced in CRC tissue samples [14]. In addition, increased content of C24:1-Cer in cancer tissue with lymph node metastases has been found, which may also affect the high variability of ceramide levels depending on the advancement of the cancer. Currently, it is known too, that large amounts of ceramides are generated when colon cancer cells are additionally affected by stressors, for example, low nutrients or hypoxia, consequently leading to rapid apoptosis in a dose-dependent manner [15]. Observations from the laboratory may soon find practical use. Combination therapy with synthetic C6:0-Cer and chemotherapeutic drugs raised the effectiveness of treatment in cancer cell lines [16]. 

The complexity of ceramide metabolism is enormous, and there is still a lot of conflicting data on its regulation. In the present study, we measured the protein level of the major enzymes responsible for de novo ceramide synthesis. It was found that some enzyme content in CRC tissue was lower than in normal intestinal tissue, for SPT, CerS1, and CerS5. This result is consistent with the observations of some authors who discovered decreased ceramide synthase expression in a human colorectal carcinoma cell line compared with another cell type [3]. On the other hand, other authors found increased expression of ceramide synthases in CRC tissue [14]. However, in both cases, changes in ceramide levels were similar to our study, which further emphasizes the importance of its accumulation within colorectal cancer.

CerS exhibit different preferences for substrates and thus produce distinct ceramides with unique fatty acids with specific properties. It has been shown that overexpression of some CerS species induced the death of colon cancer cells in vitro, whereas overexpression of other CerS increased proliferation of these cells. [17]. Another study showed that strong CerS5 staining correlated with poor prognosis in patients with CRC [18]. Mice deficient in one CerS species were viable and did not have obvious defects in the gastrointestinal tract, suggesting that the function of individual ceramide synthases under physiological conditions can complement each other [19]. 

Cytochrome c oxidase (COX or complex IV) dysfunction increases cellular ceramide levels mainly mediated via the activity of ceramide synthase [20]. Changing the level of ceramides acting as a chemical messenger in cells usually stimulates apoptosis. Studies on assessing the COX level in CRC are scarce. Zhang et al. demonstrated the significantly increased COX expression in CRC tissue when compared to adjacent nontumor tissues using western blot analysis [21]. We found a different relationship in our study, so this issue requires further investigation. 

We have not discovered why ceramide levels increased in CRC patients but the ceramide synthase expression levels decreased. The most likely explanation would be a compensatory mechanism of downregulating the expression of enzymes involved in de novo ceramide synthesis, such as SPT and CerS, under the influence of elevated ceramide levels. This suggestion seems to be confirmed by the results of the study by Mullen et al. [22] who demonstrated that the targeted knockdown of CerS1 and CerS5 did not cause the expected robust decreases in their specific products, C18:0-Cer and C16:0-Cer, respectively. In addition, there were few unanticipated increases in several sphingolipid classes, reflecting the complex metabolism of sphingolipids in the cell. For example, the downregulation of CerS1 upregulated several ceramide species, which highlights the complexity of ceramide metabolism regulation processes. Our study was conducted on colorectal cancer tissue, which is more heterogeneous compared to cell cultures and more difficult to interpret.

It should be remembered that ceramide levels in the cell is the result of its synthesis and degradation. However, the contribution of these two processes to maintaining a similar amount of intracellular ceramide can be quite different in distinct cases. The plasma ceramide concentration is not associated directly with the ceramide amount in cancer tissue, and apoptotic cells that release intracellular ceramides (at cell lysis) to the tumor microenvironment are considered the main (except immune cells) likely source of ceramides in cancerous tissue. The reason for the change in ceramide plasma levels in colorectal cancer patients is unknown, and it is not known if this is due to their altered production or consumption, or both. Literature data showed the accumulation of ceramides also in other cancer cells. For example, the level of total ceramides in breast cancer tissue was proved to be higher than in peri-tumor tissue [11], and the levels of C16:0-Cer, C24:1-Cer, and C24:0-Cer were significantly elevated in breast cancer tissue as compared with normal breast tissue [23]. As in our study, the highest contents in squamous cell carcinomas of the head and neck (HNSCC) were found for C16:0-Cer and C24:1-Cer, in turn, Karahatay et al. showed that C16:0-Cer, C24:0-Cer, and C24:1-Cer levels were significantly increased, while C18:0-Cer and C18:1-Cer levels were significantly reduced in HNSCC compared to normal tissue. However, because some other studies have shown reduced or increased ceramide content in tumors, it appears that molecular mechanisms regulating sphingolipid levels may be specific for the different histological types of the tissue [11,24]. 

Laboratory tests on a human colon cancer cell line confirmed that the administration of exogenous C16-Cer promoted programmed cell death, and it was suggested that maybe an increase of endogenous production of C16-Cer may also exert similar effects [25]. However, the biological activity of the same ceramide analogs in different types of histological tissues may be completely different. In the HNSCC cell line, C16:0-Cer had antiapoptotic properties [26], whereas in HeLa cells, C16:0-ceramide worked as a proapoptotic factor [27]. Additionally, ceramides with specific chain lengths can affect the fate of the same cancer cells differently. Long-chain ceramides and very-long-chain ceramides have shown the opposite effect on human colon cancer cell line (HCT-116) growth [28]. In addition, in HNSCC cell lines, C16:0-Cer has antiapoptotic properties, while C18:0-Cer has proapoptotic features [26]. Moreover, the deficiency of some ceramides may be compensated for by increased expression of others, resulting in an altered synthesis of different ceramide analogs. 

The different amounts of individual ceramide analogs affect cell signaling, and the changed quantitative balance between ceramides of various chain lengths seems to play a pivotal role in cancer progression [29]. Attenuation of C18:0-Cer in HNSCC significantly correlated with cancer advancement presented as a lymphovascular spread [29]. However, in our population of patients with more advanced colorectal cancer, what is most interesting, a higher content of C20:0-Cer and C24:1-Cer was observed in the tumor. Similar changes were found in other tissues by some authors. In pancreatic cancer patients, Jiang et al. found a significant change in the sphingolipid profiles depending on the grade of the neoplasm; a higher amount of C16:0-Cer and C24:1-Cer was found in pancreatic cancer tissue with positive regional lymph node metastases than in patients without metastases [30]. 

The authors suggested that ceramides do not leak out from the tumor into the systemic circulation. Short-chain ceramides (C2:0-ceramide and C6:0-ceramide) are water-soluble and cell-membrane permeable and can easily leave the cell [31]. In contrast, long-chain and very-long-chain ceramides are released as extracellular vesicles from platelets, immune cells, and other tissues, but it is still unknown what cells or tissues are the main sources of plasma sphingolipids in patients with CRC [32]. 

In our study, we also measured the blood sphingolipid profile in patients with varying stages of colorectal cancer. The share of individual species in the entire ceramide pool was different in plasma, colorectal cancer tissue, or surrounding normal intestinal tissue. By far the highest-circulating levels were found for C24:0-Cer (47.39% of total ceramide) and C24:1-Cer (27.91% of total ceramide). A somewhat smaller but still significant concentration in the plasma was demonstrated for C22:0-Cer, C16:0-Cer, and S1P. Similar results were obtained by other researchers. According to Haus et al., the major ceramides in plasma were C24:1-Cer and C24:0-Cer [33]. Dubois et al. found that plasma ceramide levels were higher in CRC patients than in healthy donors, and the most abundant plasma levels were C24:0-Cer (44.39%), C24:1-Cer (23.74%), C22:0-Cer (16.06%), and C16:0-Cer (7.51%) [15]. It was also observed that the total plasma ceramide level was considerably increased over basal values in patients with a complete response and partial response to radiotherapy, remained unchanged in patients with stable disease, and decreased significantly in patients with progressive disease [15]. Total ceramide levels before therapy were not indicative of tumor response, but the authors concluded that circulating ceramide levels may be useful as an early biomarker of tumor response to treatment. Apart from that, the observed increases in total ceramide levels were not related to patient age, sex, smoking status, number of metastases, or tumor location and volume [15,34]. It is not known whether the populations studied so far have also been evaluated for diabetes and hypoglycaemic therapy, which may affect the results and total ceramide compared to healthy nondiabetic subjects [33]. It is currently known that plasma ceramides are higher in adults with diabetes and a significant decrease is observed during pioglitazone treatment [35].

We found a positive correlation between plasma ceramides and stated that only three of them (C16-Cer, C20-Cer, and C24:1-Cer) correlated statistically significantly with all other analyzed plasma ceramides (data not shown). In addition, the strongest correlation with the total plasma ceramide content was found for long-chain plasma ceramides C22:0-Cer, C24:0-Cer, and C24:1-Cer. Similar observations were made by other authors examining ulcerative colitis patients, who found that different ceramide species are in a defined ratio to each other and change significantly, making them interesting as possible biomarkers for disease control [32].

Interestingly, in the patients participating in the present study, for the first time, a higher circulating content of several ceramides was demonstrated in more advanced colorectal cancer compared to early stages lesions. The combination of four plasma ceramide concentrations (C16:0-Cer, C18:1-Cer, C20:0-Cer, C24:1-Cer) could be particularly useful for distinguishing between early and advanced CRC. The results are consistent with the observations of other authors who compared the concentrations of selected sphingolipids in the blood taken from patients with final stage CRC and healthy people. A similar plasma profile was found by Separovic et al. in patients with stage IV colorectal cancer; levels of four ceramides (C16:0-Cer, C18:0-Cer, C18:1-Cer, and C24:1-Cer) were significantly higher compared to healthy men [36]. 

The current inclusion criteria for adjuvant treatment according to TNM classification are not perfect. Only some patients qualifying for such treatment have benefit, and on the other hand, only some patients not qualifying for adjuvant treatment have further disease progression. Sometimes, patients with stage II colorectal cancer have poorer survival than patients with stage IIIA. 

The current work is only a preliminary study with limited sample size and performed in a single medical center, but our discovery may soon find a practice application for a better classification of colorectal cancer. First, the finding that CRC grading can affect the ceramides profilecould be a factor driving a personalized therapeutic approach influencing start adjuvant therapy decisions or a high-risk feature to stratify patients that will benefit most from adjuvant chemotherapy inclusion. Second, the ceramide profiles in plasma could be potentially useful as a non-invasive criterion of tumor staging, a prognostic indicator of outcome, and an early marker of treatment effectiveness or disease relapse. 

The results of previous studies and our research prove that sphingolipid metabolism is extremely complex, heterogeneous at various levels of carcinogenicity, and not yet fully understood. There is a need for large trials to verify the clinical significance of ceramide profiles in CRC patients.

## 5. Conclusions

Our analysis showed a different qualitative and quantitative ceramide profile within the colorectal cancer tissue compared to healthy intestinal tissue. 

We revealed progressive and unequivocally increased amounts of some ceramide species in colorectal cancer tissue in line with the rise of the colorectal cancer stage according to TNM classification. 

Moreover, we proved that the circulating content of at least a few species of ceramides significantly changes in patients with advanced colorectal cancer and found the optimal cut-off value for this stage. It is likely that several ceramides in the plasma may be useful biomarkers to diagnose, more accurately determine staging, or control cancer progression or treatment effects, e.g., recurrence after radical surgical resection; further research is needed in this area.

## Figures and Tables

**Table 1:** Demographics and clinical characteristics of colorectal cancer (CRC) patients.

Characteristics	No. of Patients
Age at diagnosis [y]	
<60	9
60–69	19
70–79	9
80v90	8
Sex	
Males	33
Females	12
Tumor localization	
Sigmoid colon	10
Rectum	14
Cecum	6
Other parts of colon	15
Tumor size [mm]	
<30	12
31–60	20
61–90	11
>90	2
Tumor grading	
G1	2
G2	37
G3	6
Tumor staging	
TNM I	14
TNM II	10
TNM III	15
TNM IV	6

**Table 2:** Content of sphingolipids in normal colorectal tissue and colorectal cancer tissue. Statistical significance is marked with an asterisk.

Compound	Normal Tissue [pmol/mg]		Tumor [pmol/mg]	
	M ± SE	Min–Max	M ± SE	Min–Max
Sph	2.37 ± 0.19	0.35–5.79	6.38 ± 0.67 *	0.57–18.64
S1P	0.02 ± 0.00	0.00–0.08	0.05 ± 0.01 *	0.01–0.17
SPA	0.72 ± 0.09	0.10–3.10	1.48 ± 0.15 *	0.27–4.15
C14:0-Cer	1.15 ± 0.09	0.14–2.75	1.60 ± 0.11 *	0.28–2.99
C16:0-Cer	104.05 ± 7.42	7.57–195.65	120.81 ± 8.79	18.24–248.21
C18:1-Cer	0.30 ± 0.02	0.04–0.66	0.31 ± 0.02	0.09–0.74
C18:0-Cer	4.07 ± 0.27	0.39–8.97	3.09 ± 0.19 *	1.07–5.50
C20:0-Cer	1.33 ± 0.09	0.51–2.38	0.88 ± 0.07 *	0.32–1.92
C22:0-Cer	3.17 ± 0.15	1.27–4.75	3.08 ± 0.20	1.16–5.43
C24:1-Cer	14.06 ± 0.79	4.66–25.86	14.00 ± 0.84	6.51–26.12
C24:0-Cer	4.77 ± 0.25	1.21–8.09	6.55 ± 0.47 *	1.27–14.16
Totoal Cer	132.90 ± 8.42	18.01–231.51	150.34 ± 9.72	34.14–286.37

Values are mean [pmol/mg] ± standard error of mean [M ± SEM]; * *p* < 0.05 vs. normal tissue.

**Table 3:** Content of sphingolipids in colorectal cancer tissue of patients divided into groups depending on the 8th edition of the TNM classification.

Tumor	TNM I	TNM II	TNM III	TNM IV	TNM I+II	TNM III+IV
Sph	7.17 ± 1.34	6.69 ± 1.11	5.23 ± 1.05	4.25 ± 0.79	6.93 ± 0.85	4.92 ± 0.75
S1P	0.04 ± 0.01	0.06 ± 0.01	0.05 ± 0.01	0.04 ± 0.01	0.05 ± 0.01	0.04 ± 0.01
SPA	1.56 ± 0.37	1.45 ± 0.23	1.40 ± 0.27	1.21 ± 0.25	1.51 ± 0.21	1.34 ± 0.20
C14:0	1.71 ± 0.16	1.51 ± 0.22	1.55 ± 0.26	1.72 ± 0.34	1.61 ± 0.13	1.61 ± 0.20
C16:0	128.98 ± 16.15	111.58 ± 17.73	126.78 ± 19.28	108.44 ± 19.33	120.28 ± 11.84	121.05 ± 14.37
C18:1	0.37 ± 0.06	0.28 ± 0.05	0.29 ± 0.04	0.29 ± 0.05	0.33 ± 0.04	0.29 ± 0.03
C18:0	2.87 ± 0.35	2.70 ± 0.31	3.61 ± 0.34	3.32 ± 0.70	2.79 ± 0.23	3.52 ± 0.31
C20:0	0.73 ± 0.13	0.82 ± 0.14	1.02 ± 0.11	1.04 ± 0.14	0.78 ± 0.09	1.03 ± 0.09 *
C22:0	2.90 ± 0.44	2.75 ± 0.34	3.36 ± 0.35	3.62 ± 0.56	2.83 ± 0.27	3.44 ± 0.29
C24:1	12.66 ± 1.64	14.28 ± 2.07	14.96 ± 1.29	15.16 ± 1.76	13.47 ± 1.30	15.02 ± 1.01 *
C24:0	6.89 ± 1.02	6.08 ± 0.82	6.86 ± 1.04	6.27 ± 0.89	6.48 ± 0.64	6.67 ± 0.75
Total Cer	157.12 ± 17.27	140.00 ± 20.12	158.44 ± 21.08	139.85 ± 23.32	148.56 ± 13.05	152.63 ± 15.94

Values are mean [pmol/mg] ± standard error of mean [M ± SEM]; * *p* < 0.05 vs. TNM I+II and TNM III+IV.

**Table 4:** Concentration of sphingolipids in the plasma of colorectal cancer patients divided into groups depending on the 8th edition of the TNM classification.

Plasma	TNM I	TNM II	TNM III	TNM IV	TNM I+II	TNM III+IV
Sph	11.49 ± 1.22	12.51 ± 1.88	12.66 ± 1.14	14.43 ± 2.83	11.95 ± 1.06	13.05 ± 1.06
S1P	332.31 ± 22.30	365.10 ± 38.57	390.59 ± 31.41	399.46 ± 83.57	347.07 ± 20.97	392.56 ± 29.35
SPA	4.53 ± 0.34	5.09 ± 0.60	5.18 ± 0.49	4.73 ± 0.48	4.78 ± 0.33	5.08 ± 0.39
C14:0	12.24 ± 1.12	11.60 ± 1.05	12.86 ± 1.22	12.17 ± 0.25	11.95 ± 0.76	12.71 ± 0.94
C16:0	398.56 ± 45.61	407.09 ± 36.14	527.46 ± 49.80	503.25 ± 75.97	402.40 ± 29.17	522.08 ± 41.32 *
C18:1	5.61 ± 0.28	5.91 ± 0.43	7.48 ± 0.98	7.32 ± 1.10	5.74 ± 0.24	7.44 ± 0.79 *
C18:0	47.02 ± 7.21	45.06 ± 4.09	57.15 ± 5.84	59.39 ± 15.94	46.14 ± 4.27	57.65 ± 5.50
C20:0	47.26 ± 4.99	44.25 ± 3.42	56.54 ± 3.48	56.12 ± 14.70	45.91 ± 3.09	56.45 ± 3.96 *
C22:0	700.69 ± 81.36	694.71 ± 95.31	777.00 ± 75.71	656.47 ± 38.92	698.00 ± 60.31	750.22 ± 60.14
C24:1	1259.4 ± 92.4	1429.5 ± 165.5	1644.1 ± 164.3	1484.2 ± 153.7	1335.9 ± 89.6	1608.6 ± 131.3 *
C24:0	2465.7 ± 226.2	2369.7 ± 308.7	2634.5 ± 274.7	2272.9 ± 258.5	2422.5 ± 181.6	2554.1 ± 220.9
Total Cer	4936.5 ± 410.5	5007.8 ± 569.3	5717.1 ± 519.1	5051.9 ± 172.9	4968.6 ± 332.1	5569.2 ± 407.4

Values are mean [pmol/mL] ± standard error of mean [M ± SEM]; * *p* < 0.05 vs. TNM I+II and TNM III+IV.
